# ‘Foreigners’, ‘ethnic minorities’, and ‘non-Western allochtoons’: an analysis of the development of ‘ethnicity’ in health policy in the Netherlands from 1970 to 2015

**DOI:** 10.1186/s12889-017-4063-8

**Published:** 2017-01-31

**Authors:** Alana Helberg-Proctor, Agnes Meershoek, Anja Krumeich, Klasien Horstman

**Affiliations:** Department of Health, Ethics and Society, CAPHRI Care and Public Health Research Institute, Maastricht University, PO Box 616, Maastricht, 6200 MD The Netherlands

**Keywords:** Ethnicity, Ethnic minorities, Migrants, Health policy, Co-production, Race, The Netherlands

## Abstract

**Background:**

The Netherlands, because of the sustained and systematic attention it paid to migrant and minority health issues during the last quarter of the twentieth century, has been depicted as being progressive in its approach to healthcare for migrants and minorities. Recently, however, these progressive policies have changed, reflecting a trend towards problematising issues of integration in order to focus on the responsibilities that migrants and ethnic minorities bear in terms of their health. This article explores these shifts and specifically the development of particular categories of ethnicity, and examines the wider consequences that have arisen as a result.

**Methods:**

The analysis presented here entailed a qualitative content analysis of health policies for migrants and ethnic minorities from 1970 to 2015, and examined various documents and materials produced by the institutions and organisations responsible for implementing these healthcare policies during the period from 1970 to 2015.

**Results:**

Four distinct periods of political discourse related to health policy for migrants and ethnic minorities were identified. These periods of political discourse were found to shape the manner in which ethnicity and various categories and representation of *foreigners*, later *ethnic minorities*, and at present *non-Western allochtoons* are constructed in health policy and the implantation practices that follow. At present, in the Netherlands the term allochtoon is used to describe people who are considered of foreign heritage, and its antonym autochtoon is used for those who are considered native to the Netherlands. We discuss the scientific reproduction and even geneticisation of these politically produced categories of autochtoon, Western allochtoon, and non-Western allochtoon—a phenomenon that occurs when politically produced categories are prescribed or taken up by other health sectors.

**Conclusions:**

The categories of autochtoon, Western allochtoon, and non-Western allochtoon in the health sciences and the field of ethnicity and health in the Netherlands today have been co-produced by society and science. Policy formulated on the basis of specific political discourse informs the conceptualisations about groups and categories, issues, and solutions, and when these are institutionalised in subsequent health policy, databases, research, and care practices, these ethnic categorisations are replicated in a manner that renders them ‘real’ and enables them to be applied both socially and scientifically, culminating in pronouncements as to who is the *same* and who is *different* in Dutch society and science.

## Background

The Netherlands has been portrayed as being progressive in terms of the sustained and systematic attention it has paid to migrant and minority health initiatives introduced during the last quarter of the twentieth century [1–3]. During this period, various organisations and initiatives received national funding and support to improve the health and healthcare of migrants and ethnic minorities in the Netherlands; as a result, various different services became available. In this article, however, we focus on another issue as we analyse the impact of these policies—namely the manner in which they act to produce specific categories of ethnicity and of sameness and difference.

Globally, many different categorisation systems are used in health policy and health research in relation to the topic of ethnicity and race. Because of widely documented ethnic health disparities and differences, it is considered relevant to include ethnicity in health research in order to investigate the causes of and possible solutions to ethnic health inequalities. The field of ethnicity and health is a field of research and scholarships specifically dedicated to investigating these ethnic health inequalities and differences relevant to health, and the role of ethnicity in all aspects of health and health care and policy. Various scholars, however, have warned that the use of ethnicity in health research and policy is a sensitive topic, with issues related to privacy and the potential misuse of information against the interests of ethnic groups often mentioned as causing concern [4]; in addition, potential patients who are part of an ethnic minority have expressed uncertainty and unease as to how the data may be used [5]. Furthermore, scholars have warned specifically against the application of policies that seek to standardise the categorisation of ethnicity, as these have consequences both for society and for science [6, 7]. Smart et al. [7], for instance, caution that the formation of census categories and their subsequent adoption for health research can have significant consequences, given that the widely accepted and routine use of these categories works to obscure their socio-political origins; this subsequently allows for the ‘naturalisation’ or even ‘geneticisation’ of these policy categories.

At present, in the field of ethnicity and health in the Netherlands, one can encounter specific language, concepts, categories, and populations related to ‘allochthony’, specific countries of origin, ‘Westernism’, and ‘non-Westernism’ in healthcare and research. In this article, we explore the origins of these particular categories and, specifically, the role that political discourse plays in the production of categories of ethnicity deemed biologically relevant in the Netherlands. To this end, we analyse how particular political discourses have produced categories and representations of populations; we then examined the perceived problems related to the healthcare of persons who migrated to the Netherlands during the last four decades and to their descendants that emerged from these narratives. The aim of this paper is thus twofold: Firstly, to reveal the dynamic production of categories and representations of ethnicity in health policy in the Netherlands, and secondly, to shed light on the role that these political discourses have played in the production of categories of ethnicity deemed biologically relevant in the field of health in the Netherlands today.

### Analysing health policies

Political discourses and policies can be understood as ‘diagnostic/prescriptive stories that tell, within a given issue terrain, what needs fixing and how it might be fixed’ [8–10]. Bacchi [9] states that a policy does not simply address and seek to solve a social problem; rather, it forms part of a process by which social problems are shaped, thus turning the formulation of policies into ‘problematizing activities’. In the Netherlands, such critical analyses have already generated important insights as to how policies seek to address certain ‘social problems’; how they also produce specific problematized ‘categories’; and how groups in need of some kind of policy intervention are identified [11–14]. For example, Roggeband and Verloo [11] explore how policies in relation to migrant women in the Netherlands have evolved over time, and show how specific representations of migrant women as constituting a ‘policy problem’ ultimately led to the reinforcement of social stratification in Dutch society, given that these policies implicitly create an ‘us’ and ‘them’ dichotomy:

The specific framing in Dutch policies creates and reproduces social dichotomies and oppositions between Dutch and ‘others’, between men and women, and between traditional (Muslim) and modern (‘Western’) cultures. […] The negative representations of migrant women as traditional, backward and (potentially) as victims may limit the discursive opportunities for identification and participation of migrant women, and thus may have the opposite effect from what government aims to accomplish [11].

Roggeband and Verloo draw on the work of Snow and Benford [15] to inform the central elements of their analytical framework, namely the *diagnosis* (how a problem is defined), *prognosis* (what the proposed solution to the problem is), and *call for action* (who is responsible for solving the problem). Our analysis adopts a comparable approach, similarly attending to ‘diagnosis’, ‘prognosis, and ‘call for action’ in order to understand how problems are represented in health policy; we also focus on the specific ‘categories’ included in these representations.

## Methods

The analysis presented here was carried out in several steps. First, formative research was conducted to identify the specific periods and political discourses relevant to the formulation of health policies geared towards groups who migrated to the Netherlands during the last four decades and to their descendants, referred to as ethnic minorities of migrants. The development of ethnic minority and migration policies in the Netherlands is generally perceived to have taken place during several distinct periods [16, 17]. To identify these distinct periods and discourses, formative research was undertaken to chronologically review all national policies relating to ethnic minorities and migration policy as of the 1970s: This review included the *Nota’s Buitenlandse Werknemers* [policy briefs on foreign employees] of the 1970s, the *Minderhedenbeleid* [minority policy] of the 1980s and early 1990s, the *Integratiebeleid Etnische Minderheden* [integration policy ethnic minority] of the later 1990s and early 2000s, and the *Integratiebeleid* [integration policy] of the 2000s to the present. Because the research and analysis presented in this article focusses specifically on national health policies, after reviewing the above listed national ethnic minorities and migration policy between 1970 and 2015, the sections in these national policies concerning specifically healthcare were analysed to identify the different periods and discourses related to health policy for migrants and ethnic minorities. From this formative research, we identified four distinct periods and discourses related to health policy for migrants and ethnic minorities; it should be noted that these four periods correspond to the periods described in previous research [16, 17].

Each of the first three of these periods was initiated by a shift in policy that marked a change in political discourse, announced in three crucial introductory documents— the *Brief on Foreign Workers* of 1970, the *Minority Policy Memorandum* of 1982, and the *Concept Integration Policy Ethnic Minorities* of 1994. In addition to these three national policy documents, we also included in the analysis various documents produced by the national institutions and organisations responsible for deploying these policies in the area of healthcare during these periods (such as the Consultation Body Medical Care Foreign Employees, Bureau Health Education Healthcare for Foreigners, and National Institute for Health Promotion and Disease Prevention). Relevant to and illustrative of the fourth and last period that was analysed is the cessation of previously implemented policies and subsidies, and the initiation of a new direction in policies related to the field of ethnicity and health; for this fourth period the primary sources analysed include transcripts of parliamentary discussions and official parliamentary letters and announcements. In total 21 primary sources were included in the analysis (see Appendix 1 for the list of all primary sources).

Having identified the relevant policy documents and additional primary sources for each of the four periods, a second round of analyses were conducted to investigate the *diagnosis* (how a problem is defined), the *prognosis* (what the proposed solution to the problem is), and a *call for action* (who is responsible for solving the problem) present in these policies, and to investigate the categories and representations that emerged during each of these periods. During this second round of analyses, the documents described above were re-evaluated to identify the diagnoses, prognoses, and calls for action present in these policies, along with some of the implementation practices they led to. Lastly, during this second round of analyses, we also sought to distinguish and analyse the categories used and representations made of those people who migrated to the Netherlands during the last four decades and of their descendants.

## Results

### The politics and practices of primary health care for foreigners, ethnic minorities, and non-Western allochtoons

#### Health policy and care for foreigners: a temporary employment agenda

From the mid-1950s up until the mid-1970s, people from various countries, including Italy, Spain, Portugal, Turkey, Greece, Morocco, former Yugoslavia, Tunisia, and the former Dutch Antilles were recruited to come to work in the Netherlands [18, 19]. This period also saw the migration of people from Suriname, a former Dutch colony, as well as ‘repatriates’^1^ from the former Dutch East Indies during the 1950s, Mollukens, and refugees from Eastern-Europe, Vietnam, and Latin-America to the Netherlands [19]. Despite this influx of people migrating to the Netherlands, those in political power during this time did not view the Netherlands as a country of immigration [18]. For instance, in the ministerial *Memorandum on Foreign Workers* of 1970 [18] it is unequivocally stated that the Netherlands was ‘not an immigration country’, and thus foreign workers were expected to eventually return to their countries of origin. Regarding the preservation of ‘own identity’, the *Memorandum on Foreign Workers* of 1970 states:

Regarding foreign employees who generally will only remain in our country for a short period, the emphasis will be placed on the maintenance of own identity. Readjustment difficulties upon return to their own country will this way remain as minimal as possible [18]. (authors’ translation; see Appendix 2. item A. for the original Dutch text)

What the political discourse and policies during this first period embed is the production of a specific category of *foreign workers* who are recognised as being culturally and linguistically different; however, these differences were at the time not seen as being problematical because the maintenance of ‘own identity’ was deemed to be important in order for the foreign worker to eventually return home. This narrative of the Netherlands not being an immigration country had implications for the management of primary health care for these foreign employees.

Initially, the occupational doctors first came in contact with the new foreign employees, and soon various professional publications appeared on the specificities of treating employees who came from outside the Netherlands [20]. Taking stock of these concerns, in 1972 the then Minister of Public Health and Environmental Hygiene established the *Overlegorgaan Medische Verzorging Buitenlandse Werknemers* (OMVBW) [Consultation Body Medical Care Foreign Employees] in order to accomplish the following: to find solutions to the problems which occur in the medical care of foreign employees, to help implement these solutions, and finally, to advise the Minister concerned as to how to proceed. In 1974, in their first advisory document, the OMVBW reached four specific conclusions about the problems which occur in the medical care of foreign employees; of these, language and communication problems were described as the ‘biggest problem’ in the medical care of foreign employees [20]. Subsequently, based on this finding that language and communication are the primary cause of problems in providing care for foreign employees, the OMVBM initiated and set up in 1976 the *Bureau Voorlichting Gezondheidszorg Buitenlanders* (BVGB) [Bureau Health Education Healthcare for Foreigners].

In 1980, the BVGB published one of its first booklets entitled *De buitenlandse patient* [The foreign patient] for healthcare professionals who came into contact with foreign employees as their patients [21]. The booklet aimed to increase a care provider’s background information of their foreign patients to ‘prevent unnecessary irritation’ and ‘increase understanding about their foreign patients’ [21]. As such, the booklet provides information that gives the care professional insights that will ‘breed more sympathy and understanding for their foreign patients’ [21]. Additionally, the booklet provides suggestions as to how care providers can better communicate with foreign patients, on how they should respond to particular situations, and encourages them to use bilingual materials available from the BVGB [21–23].

The diagnosis which emerged during this time identified the problem as the unfamiliarity of the Dutch care provider with their foreign patients and the communication problems that exist due to linguistic and cultural differences. It follows that the prognosis (the proposed solution to the problem) was, as with all public provisions produced for these groups during this time [16] to accommodate and guide foreigners through the healthcare system during their time in the Netherlands—which was deemed achievable through the education of care professionals. Because migrants were expected to eventually return to their countries of origin, ‘maintenance of culture’ and preservation of ‘own identity’ was considered desirable, as this would facilitate the eventual return of foreign employees to their homelands. For this reason, the focus of the diagnosis and prognosis during this period was on changes to be made by Dutch care providers and not by the foreign patients themselves.

#### Health policy and care for ethnic minorities: a multicultural agenda

By the early 1980s expectations regarding the return of foreign workers to their homelands had changed drastically, and along with it, the political discourse had shifted. An important 1979 report entitled *Etnische minderheden* [Ethnic minorities] from the Scientific Council for Government Policy was the first significant acknowledgement of the fact that persons who migrated to the Netherlands as temporary foreigners were now permanent residents, and the report served as a catalyst into a new period of ethnic minority politics [17, 19]. This new political period would come to focus on the need for the structural improvement of minorities’ social and economic position within the newly conceptualised multi-ethnic Dutch society [24]. What emerges from the political discourse and policies during this period is the production of the specific category labelled *ethnic minorities*, which refers to people who are socially and economically disadvantaged and culturally and linguistically different. This difference was, again, however, not problematised per se in the policies of this period, as the prevailing view was that it was important to aid ethnic minorities to find their way to public services in order to have equal access to public provisions [25].

Reflecting the shift in government policy, two primary institutions, which had been originally created to facilitate the provision of medical care for patients who were previously called foreigners, were renamed, replacing the word ‘foreigners’ in their titles with the word ‘minorities’. Thus, the *Bureau Voorlichting Gezondheidszorg Buitenlanders* became the *Bureau Voorlichting Gezondheidszorg Minderheden* [Bureau Health Education Healthcare for Minorities], and the *Overlegorgaan Medische Verzorging Buitenlandse Werknemers* became the *Overlegorgaan Medische Verzorging Minderheden* [Consultation Body for Medical Care of Minorities]. Their new primary task was to increase the *accessibility* of Dutch healthcare services and to ensure equal access to public service for all minority groups [25]. The *Minority Policy Memorandum* of 1982 states:

Also with regard to healthcare, the starting point is that members of ethnic minority groups should have equal access to provisions […]. The means that for the accessibility of services, it is desirable that the expertise of the care providers in relation to this patient group is increased. For this purpose the Ministry of Wellbeing, Health and Culture has developed a programme containing the following elements: Firstly, in 1982, 35,000 copies of the bulletin *Gezondheidszorg voor de buitenlandse patiënt* [Healthcare for the foreign patient] were distributed among doctors, midwives, community nurses, and healthcare facilities [25]. (authors’ translation, see Appendix 2. item B. for original Dutch text)

Thus, while the political discourse shifted significantly—from a discourse about (temporary) foreign workers to a discourse about (permanent) ethnic minorities in Dutch society—the prognoses and calls to action did not. Firstly, the diagnosis continued to focus on communication problems caused by linguistic and cultural differences; secondly, the responsibility and call for action to improve these issues was again placed on the healthcare provider, for whom ‘expertise needed to be increased’ [25].

Two central themes can be identified in the area of primary care related to ethnic minorities: firstly, the necessity to provide general information and training for all healthcare providers (what today would be known as the interculturalisation of health care); and secondly, the development of group and individual health education in minority groups’ own language and culture. Light can be shed on the interculturalisation of healthcare by examining the bulletin *Gezondheidszorg voor de buitenlandse patiënt* [Healthcare for the foreign patient] distributed among physicians, nurses, midwives, and health centres in the Netherlands, as this was one of the first elements introduced to ensure equal access to healthcare for ethnic minorities in the first Minority Policy Memorandum in 1982 [25, 26].

The bulletin starts with a definition of foreign patients and states that this term is used to describe patients who belong to ethnic minorities; it then provides a table with the key statistics in regard to this population organised by country of origin, namely Greece, Italy, (former) Yugoslavia, Portugal, Spain, Turkey, Morocco, and Indonesia [26]. The introduction states that while ‘in principal every employee of the Dutch healthcare system can encounter the responsibility of caring for a foreign patients’ many care providers consider themselves ‘ill-equipped to care for these patients whose language, culture, habits, and health problems they are barely or not at all familiar with’ [26]. And so, the bulletin offers information on these cultures, habits, and health problems in order to better equip care providers to care for their ethnic minority patients. Similarly, further materials developed by the BVGM in subsequent years were formulated with the explicit aims of complying with the discourse about multiculturalism. For example, the workbook *Voorlichting voor migranten, een methodisch werkboek* [Health education for migrants, a methods workbook], is a workbook designed for instructors working with Dutch care providers who are being trained to offer health education to migrants [27]. This workbook states that it aims to ‘create acceptance of a multi-cultural society, with all of its advantages and disadvantages’, and seeks to achieve this aim by providing information on ethnic minorities’ culture, on the effects of migration, and by discussing the willingness to ‘recognise multiculturalism’—the acceptance of which can be tested by practising respectful and non-judgmental communication skills included in the book [27]. In addition to the information provided to healthcare providers, during this political period we also see the further development of group and individual health education programmes in the migrant groups’ own language and culture [‘voorlichting eigen taal en cultuur’].

Thus, while the political discourse changes from the 1970s to the 1980s, the focus and responsibilities of the interventions (prognosis and call for action) in health services provided for foreign employees in the 1970s and ethnic minorities and migrants in the 1980s did not differ much: Health providers formed the primary *targets* of these educational interventions prescribed by policy during both periods. Furthermore we see similarities, among the first and second political discourses with regard to the populations considered to be the object of these concerns. Although these populations were first produced as temporary foreign employees, and later as permanent ethnic minorities, the actual countries of origin of the persons deemed to belong to these categories were similar—mostly Turkey, Morocco, China, Portugal, Italy, Spain, Greece, [former] Yugoslavia, Suriname, and the Dutch Antilles.

#### Health policy and care for ‘allochtoons’: an integration agenda

Towards the late 1980s and early 1990s, ethnic minority policies and multicultural discourse experienced significant and increasing criticism from within politics, as well as from academia in the Netherlands [16, 17, 28, 29]. These criticisms pointed to the continued social and economic deprivation of minorities, and called for a revision of current policies. In 1994, the government responded to these concerns in the *Contourennota Integratiebeleid Etnische Minderheden* [integration policy ethnic minorities], in which it is stated that, although the government had achieved some significant improvements through the implementation of its minority policies (especially in regard to specific areas such as housing, education, and work), too little progress had been made under the minority policies [30]. For this reason, the government adopted a ‘new vision on the presence of persons from diverse cultures in the Netherlands’. The *Contourennota Integratiebeleid Etnische Minderheden* of 1994 states:

As a guiding principle for a new vision on the presence of persons from diverse cultures in the Netherlands—regardless whether they are newcomers or have been in the Netherlands longer—the cabinet adheres to the concept of citizenship […]. Citizenship implies for all those involved in the integration process a choice for sustained participation in Dutch society, with all the associated right and obligations [30]. (Authors’ translation, see Appendix 2. item C. for original Dutch text)

During this political period there is a shift in the language used to describe ethnic minorities in national healthcare policy, namely the word *allochtoons* is now used to describe those groups previously referred to as foreign workers and ethnic minorities. In English, the word *allochthonous* is used in the field of geology and literally means that something originated or was formed somewhere other than where it is found. In the Netherlands the word *allochtoon* is used to describe and categorize individuals and groups who are considered of foreign heritage, and its antonym *autochtoon* is used to describe and categorize individuals and groups who are considered native to the Netherlands. Furthermore, during this political period, with its focus on citizenship, integration, and participation, a shift took place from a diagnosis that stated that communication problems between foreign workers and Dutch care professionals and unequal access for ethnic minorities were the source of the problems, towards a focus on the cultural problems allochtoons brought to the health and healthcare situation.

In 1995, the then Minister of Health, Welfare, and Sport (VWS), Mrs Borst-Eilers, addressed the form in which the new integration policies would take with regard to healthcare for allochtoons. In this policy letter, the Minister states that the health situation of allochtoons gives reason for concern, and defines these differences, when compared to the Dutch population, as resulting from ‘socio-economic and, specifically, cultural-adjustment problems’ [31]. This shift in discourse, diagnosis, and language, with the use of this specific term allochtoons, is critical as it indicates a shift in how the population which these health policies seek to target is constructed in the discourse of this new period. Namely, whereas in the previous two discourses, we observed policies and programmes geared towards foreign employees and later towards ethnic minorities from Turkey, Morocco, Portugal, Italy, Spain, Greece, (former) Yugoslavia, China, Indonesia, Suriname, and the (former) Dutch Antilles, the category allochtoons (as included in the health policy letter of 1995) only mentions the categories Turkish, Moroccan, Surinamese, and Antillean. In 1999, this demarcation and differentiation was formalised by the Central Bureau of Statistics (CBS), when certain countries of origin, such as Turkey, Morocco, China, Suriname, and the (former) Dutch Antilles, were defined as *non-Western*, and other countries of origin, such as Italy, Japan, Spain, Portugal, Greece, and Indonesia, were referred to as *Western* [32]. This led to whole populations being labelled as either Western allochtoons or non-Western allochtoons. The justification for this distinction and division was based on perceived socioeconomic and cultural *similarities* and *differences*, where people originating from Europe (excluding Turkey), North-America, Oceania, Japan and Indonesia were considered to be similar to the Dutch with regard to ‘socioeconomic and cultural position in Dutch society’, and those with origins in Africa, Asia (excluding Japan and Indonesia), Latin-America, and Turkey were considered to be decidedly different from Dutch society and other Western groups [32]. This discourse had implications for the management of primary healthcare for these so called non-Western allochtoons.

During this period, the diagnosis of the problem and the prognosis shifted from the accommodation of minorities in a multi-ethnic society and in the healthcare system, to a problem definition that pointed to the lack of integration and participation of minorities, which included cultural problems as a focal determinant of health inequalities. Particularly, this shift can be seen in the categories produced in these representations, from foreign employees and ethnic minorities to non-Western allochtoons, with a specific characterisation of this category as having specific cultural problems affecting health and healthcare. As such, it follows that specific interventions were deemed necessary for this particular group to address the cultural and integration-related problems affecting their health; one such widely employed intervention is the *allochtone zorgconsulent* [allochtonous care consultant].

By the late 1990s and early 2000s the BVGM had trained and certified health educators to give health education to minority groups in their own language and culture [voorlichting eigen taal en cultuur] (VETC), and by 2002, twenty regional and local support centres had been set up to assist VETC programmes across the country. By the late 1990s and early 2000s, VETC consultants were increasingly referred to as allochtone zorgconsulenten [allochthonous care consultants], and in 2002, this shift in terminology was solidified with the publication of the handbook *De allochtone zorgconsulent* [33]. The handbook was produced in a collaboration between various national organisations, including Forum Instituut voor Multiculturele Vraagstukken [institute for multicultural affairs], The Netherlands Organisation for Health Research and Development, and National Institute for Health Promotion and Disease Prevention (NIGZ). The NIGZ was founded in 1996, when the Bureau Health Education Healthcare for Minorities merged with four other organisations in order to become a national institute. In the first chapter of this handbook, in the paragraph titled ‘Een interactief antwoord op huidige knelpunten’ [An interactive answer to current bottlenecks], problematic ‘bottlenecks’ were identified as all relating to the reciprocal lack of information on the part of ‘allochtone patients’ and healthcare providers, and ‘cultural and languages difference’ [33]. Specifically, it is stated that allochtone patients ‘often know little about the anatomy of the body, the relationship between anxiety and physical complaints, and the structure of the Dutch healthcare system’ and that ‘allochtoons very often present somatic and psychosocial problems’; care providers in turn ‘often lack the necessary knowledge of opinions and expectations of allochtone patients regarding health, (illness-related) behaviour, and treatment’ [33]. The allochthonous care consultant is perceived to be an interactive answer to these described problems, as the allochthonous care consultants can ‘bridge the differences’ between patients and care providers and ‘better inform each other about the other party’ [33].

What emerges from the political discourse, policies, and programmes during this period is thus the production of a category, non-Western allochtoons, who are deemed to be culturally and linguistically different, and this difference is problematised, in that it is considered to have negative implications for health and healthcare. For this reason, not only health professionals (as previously seen), but also *allochtonous* patients became the targets of behavioural change interventions and programming.

#### ‘New style’ health policy and care: an agenda for genetics in care and research

During the analysis of the previous period, it has been demonstrated that perceived cultural and linguistic difference of non-Western allochtoons, compared to Western allochtoons and autochtoons, has emerged as a problematic issue in healthcare. Nevertheless, public services and projects were funded by the national government during this period, and special services for the so-called non-Western allochtoons were available. During the next period, from the mid-2000s to present, we see two new directions in the healthcare policy related to ethnic minorities and migrants—namely, the *Integratiebeleid Nieuwe Stijl* [New Style Integration Policy] and health-related policies that take into consideration the genetic and biological relevance of ethnicity for research and care.

With its onset during the early and mid-2000s, the New Style Integration Policy (as the policy makers who authored the policy named it) continues the previous discourse and focuses on active *citizenship* and individual *responsibility*, yet with an even stronger emphasis on cultural adaptation to Dutch society. During this change in discourse, the coordination of integration policies moved from the Ministry of Home Affairs, where it had been for 22 years, to the Ministry of Justice under a new Minister for Alien Affairs and Integration [17]. Within this new period, intercultural care services, such as health education in a group’s own language and culture and the allochthonous care consultant were increasingly seen as obstructing integration. For instance, in 2011, the current Minister of Health (VWS) Mrs Schippers was asked the following question by parliament member Gerbrands from the Party for Freedom (a right-wing political party):

‘Do you share the opinion that appointing allochthonous care consultants send the wrong signal, namely that the medical world needs to adapt to allochthoons instead of the other way round?’ [34] (authors’ translation, see Appendix 2. item D. for original Dutch text).

Health Minister Schipper’ reply exemplifies the political discourse of the New Style period: She states:

‘I am of the opinion that healthcare contributions [premiums] should be spent providing care. Making yourself understandable is the responsibility of people who locate themselves here. If the government and institutions keep providing solutions which reduce the necessity to learn the language, in the long-run the position of the allochthoon will even be damaged’ [34] (authors’ translation, see Appendix 2. item E. for original Dutch text)

And indeed, with the onset and development of the New Style Integration Policies and the general retreat of the welfare state in the Netherlands, many institutions, projects, and programmes involved in intercultural care lost their subsidies and so, many closed.^1^

What continues to emerge from the current political discourse and policies is the image of the highly problematised non-Western allochtoons, who are culturally and linguistically different and in urgent need of integrating and adapting, as their cultural and linguistic differences are represented as having negative (and one might add, self-inflicted) consequences for their health and their access to healthcare. What is interesting, however, is that while current ‘New Style’ political discourse discourages policies and practices geared toward making healthcare more accessible and intercultural (because cultural and social integration and assimilation into Dutch society are desired), *ethnicity* is, however, being taken into account in other areas related to health policies and practices. What has emerged is a second shift in focus, away from attending to cultural and linguistic differences towards taking into consideration biological and genetic factors.

#### Registering ‘ethnicity’ for healthcare and public health research

From 2007 to 2011, the possible registration of ethnicity for healthcare and research purposes was the subject of political and public debate in the Netherlands. This discussion formally started in 2007 when the *Trendanalyse Biotechnologie* 2 [Trend analysis Biotechnology] was published, in which ethnicity is named a possible relevant factor in scientific research, genetic diagnosis, and genetic public health research. Based on this report, the then State Secretary of Health (VWS) requested the Organisation for Clinical Genetics Netherlands (VKGN) to inform her whether there was a relationship between ethnicity and genetics and, if so, what the possible consequences on health of this relationship would be [35] In their reply, the VKGN concluded that ethnicity can be seen as a relevant factor in scientific research, genetics diagnostics, and genetic public health research, because these groups are subject to migration, isolation, selection, and ‘cultural influences’, and thus genetic differences in ‘disease-causing mutations’ among ethnic groups do exist [36]. However, the VKGN authors also warn that, in practice, it is difficult to deal with ethnicity accurately as there exists no ‘generally accepted division of ethnic groups’ [36]. In 2008, upon receiving this reply from the VKGN, the then State Secretary of Health (VWS) again sent a letter to the Dutch parliament, in which she stated:

I have informed myself, by the Vereniging van Klinische Genetica Nederland (VKGN) among others, regarding the possible relevance, as presumed in the Trendanalyse, of a possible relationship between genetics and ethnicity for healthcare. The VKGN has come to the conclusion that ethnicity can be a factor in scientific research, genetics diagnostics, and genetics public health research [37]. (authors’ translation, see Appendix 2. item F. for original Dutch text).

This letter establishes the genetic relevance of ethnicity, while the ambiguity of categorising ethnicity, as put forth by the VKGN when stating that no ‘generally accepted division of ethnic groups’ exists, is lost. And so, in November of 2011 after receiving additional advice about the legal aspects of registering ethnicity in care and research from the Netherlands Centre for Ethics and Health and the Council for Public Health and Health Care [37], the Minister of Health pens her final letter on this topic to the Dutch parliament, concluding that it is legally possible to register ethnicity for health research and care when using the existing municipal registration system and electronic patient files [38]. It is interesting that, during this entire time, exactly what ethnicity actually *is* is never defined. However, the use of these particular systems (i.e. the existing municipal registration system and electronic patient files) to conduct scientific health research and for clinical care is crucial to the manner in which ethnicity is defined and co-produced as a *genetic* entity deemed scientifically and clinically relevant through these practices.

Namely, when using the existing municipal registration to conduct epidemiological and health research, researchers can link their own data and all kinds of health data available from the Central Bureau of Statistics’ StatLine database to ethnicity by matching (patient) social security numbers to the Dutch municipal registration system. In this municipal registration system, ethnicity is categorised by country-of-origin; it is further subcategorised by the Central Bureau of Statistics into the three categories: autochtoon, Western allochtoon, and non-Western allochtoon [39]. Thus, the establishment of ethnicity as a genetically relevant entity through policy formation, the subsequent possibilities for registering ethnicity in research and care, and the municipal registration system operative during the process when this policy was formulated resulted in the scientific reproduction of politically produced categories and worked in tandem to co-produce the particular categories autochtoon, Western allochtoon, and non-Western allochtoon as genetically relevant categories applicable to certain groups of people.

## Discussion

A few words need be said about the limitations of our study. The focus was on political discourse and, as such, on political and other actors involved in the institutions concerned. Therefore, the complexity of what occurred during the years discussed here was more chaotic, less linear, and more unstructured than what has been described. This occurred as a result of the following: first, although the transitions between the political episodes described included sharp breaks and shifts, a particular programme or activity might have received funding for several years, and thus it eventually ‘outlived’ the original discourse that inspired it. Secondly, the national policies discussed in our analysis, which determined the official national political agenda and discourse of a period, were the result of particular political parties being in power at those times. What is striking is that many of the elements observed in discussions, debates, and disagreements in the notes of parliamentary discussion during these times were obscured when the final national policies were formulated, and thus did not form part of our analysis here as we focused on the official national policies. Nevertheless, the analysis that has been made is still relevant and significant, as it is based on the actual policies that were implemented (regardless of the discussion that took place around them).

Thus, while the Netherlands has previously been presented as being exemplary for the sustained and systematic attention it pays to the problems of migrant health [1–3], our analysis draws attention to other consequences of the political discourses and policies that have taken place—namely the ways in which they significantly shape the categories and orders of ethnicity that are encountered in society, science, and care, particularly in the way that they make some groups culturally, socially, and biologically *different*. This discussion is especially relevant, given the move by some European Union member states to adopt and implement policies in health systems to address health inequalities among migrants and ethnic minorities [40, 41]. An examination of (health) policies related to migrants and minorities as ‘problematizing activities’ [9], which actively produce narratives of what and who is in need of changing, can reveal and provide entry points for addressing the *otherisation* through policy of already disenfranchised groups across Europe. The Dutch case analysed here offers lessons for other countries formulating health polices specially for groups considered ethnic minorities and migrants communities. Namely, while presumably unintentional, such health policies can work to reify, perpetuate and ‘spread’ into the area of healthcare politically driven notions and categories of nationalism which excluded certain groups. Specifically when used in the area of healthcare and health research the use of these might even work to reify these notions as scientifically and even genetically valid divisions of the population of a country. This is especially important in the context of health, as the social and external assignment of ethnicity to individuals, as well as the racism and exclusion which might follow from being considered and treated as not belonging in a specific politically informed nationalistic discourse might have been adverse health effects [42, 43]. Thus, paradoxically, while ethnicity is included in health research, care and policy specifically in order to combat health disparities, our analysis of the Netherlands shows that the manner which ethnicity is addressed in these practices and polices might actually be intertwined with and contribute to the very societal dynamics which produce the larger societal notions of difference and sameness which might underlay societal and health inequalities. Countries and governments looking to formulate health policies specifically for ethnic minorities and migrants must thus critically consider the categories, language and ‘problem formulation’ included in these policies as to prevent (unintentionally) contributing to processes of exclusion and otherisation.

Within the field of Science and Technology Studies (STS), the dynamics discussed in this article pertaining to how political discourses and policies shape the categories and representations of ethnicity in science and care, can be understood from the perspective of co-production [44]. Scholars in the field of STS attend to how knowledge production and scientific practices are engrained in and shaped by social context, while simultaneously observing how the social context is then embedded with (new) scientific knowledge, data, and representations [6, 44–47]. Regarding scientific knowledge of ethnicity and health, from an STS perspective, the categories and populations involved in the concept of ethnicity used in health research are conceived as being co-produced in a social context, and not something that is a result of a process taking place in a neutral and objective scientific setting. For instance, Epstein shows how the national social and political context in the United States shapes scientific practices related to ethnicity and race in health; this is done by mandating that ‘diversity’ be included in research and by prescribing the specific concepts and categories of race and ethnicity by which this can be carried out. This perceived need for ‘inclusive’ research, in which the diversity found in the American population and society is reflected and represented in science, has led to the alignment of social identities and (historically) socially assigned categories in the United States with those populations and categories deemed (biologically) relevant in medical science [6]. From the analysis presented in the article, a similar conclusion can be drawn for the Netherlands. We have demonstrated that social and political contexts inform the categories related to ethnicity, shown how these groups are represented in health care and research, and indicated that the data and knowledge produced in science and clinical practice co-produce these categories of ethnicity in a manner that enables them to be assessed as scientifically relevant.

## Conclusions

Four distinct periods and discourses related to health policy for migrants and ethnic minorities were identified. Our analysis illustrated how political discourse is implicated in the manner in which ethnicity and various categories and representations are produced in health policy and the programmes which follow. In our Dutch case, these policies and discourses shape the manner in which ethnicity and various categories and representations of foreigners, later ethnic minorities, and at present allochtoons were produced during the period studied. And indeed today, a simple internet search for ‘*etniciteit en gezondheid*’ [ethnicity and health] generates a first hit from the ethnicity and health page of the Dutch National Public Health Compass, the first sentence of which states: ‘*Allochtonen hebben minder goede gezondheid dan autochtonen*’ [‘The health of *allochthonous* people is not as good as that of autochthonous people’] [48]. As illustrated by this quote, at present, in the field of ethnicity and health in the Netherlands, specific language, concepts, categories, and populations related to *allochthony* can be encountered, as can specific countries of origin, and the concepts of Westernism and non-Westernism. However, as illustrated in our study, this matter-of-fact reality and these described populations in what is today the field of ethnicity and health in the Netherlands is actually a co-production between society and science. A co-production where political discourses inform the groups and categories, issues, and solutions that can be observed in this field. The institutionalisation of these categories and their representations during the formulation of subsequent health policy, databases, research, and care practices constitute a form of co-production in the field of ethnicity and health, in which these representations increasingly become *real*, and where they socially and scientifically categorise who is *same* and who is *different*.

## Appendix 1

### Primary sources

- Tweede Kamer, 1969–1970, kamerstuknummer 10504 ondernummer 2. Nota buitenlandse werknemers. The Hague: SDU; 1970.
- Gründemann RWM, Zwiers J. Overlegorgaan medische verzorging buitenlandse werknemers: verslag van werkzaamheden 1972–1986. Leiden: Nederlands Instituut voor Praeventieve Gezondheidszorg; 1988. Available online: http://publications.tno.nl/publication/34607069/t7VWSy/grundemann-1988-overlegorgaan.pdf.
- Gründemann RWM. Migranten, gezondheid en kontakten met de Nederlandse gezondheidszorg: een overzicht van ontwikkelingen in de periode 1975–1985, gebaseerd op onderzoekspublikaties. Leiden: Nederlands Instituut voor Praeventieve Gezondheidszorg TNO; 1985. Available online: http://publications.tno.nl/publication/34607058/L3AMRu/grundemann-1985-migranten.pdf.
- Bureau Voorlichting Gezondheidszorg Buitenlander. Voedingsgewoonten en dieetproblematiek bij Spanjaarden. Bunnik: Bureau Voorlichting Gezondheidszorg Buitenlanders; 1978. Available in print: Koninklijke Bibliotheek The national library of the Netherlands. https://www.kb.nl.
- Bureau Voorlichting Gezondheidszorg Buitenlanders. Zwangerschap in Nederland: Zwangerschapsklachten Turks. Bunnik: Bureau Voorlichting Gezondheidszorg Buitenlanders; 1978. Available in print: Koninklijke Bibliotheek The national library of the Netherlands. https://www.kb.nl.
- Bureau Voorlichting Gezondheidszorg Buitenlanders . De buitenlandse patient. Bunnik: Bureau Voorlichting Gezondheidszorg Buitenlanders; 1980. Available in print: Koninklijke Bibliotheek The national library of the Netherlands. https://www.kb.nl.
- Wetenschappelijke Raad voor het Regeringsbeleid. WRR- rapport 17: Etnische minderheden. The Hague: SDU; 1979. Available online http://www.wrr.nl.
- Tweede Kamer, 1982–1983, kamerstuknummer 16102, ondernummer 20–21. Minderhedenbeleid. The Hague: SDU; 1983.
- Geneeskundige Hoofdinspectie van de Volksgezondheid. De buitenlandse patient. Geneeskundige Hoofdinspectie; 1982. Available in print: Nationaal Archief, Den Haag, Ministerie van Volksgezondheid, Welzijn en Sport: Geneeskundige Hoofdinspectie van de Volksgezondheid, nummer toegang 2.27.5034, inventarisnummer 1150. http://www.nationaalarchief.nl.
- Bureau Voorlichting Gezondheidszorg Buitenlanders. Voorlichting voor migranten, een methodisch werkboek. Bunnik: Bureau Voorlichting Gezondheidszorg Buitenlanders; 1989. Available in print: Koninklijke Bibliotheek The national library of the Netherlands. https://www.kb.nl.
- Wetenschappelijke Raad voor het Regeringsbeleid. WRR-rapport 36: Allochtonenbeleid. The Hague: SDU; 1989. Available online http://www.wrr.nl.
- Tweede Kamer, 1993–1994, kamerstuknummer 23409, ondernummer 9. Motie-Apostolou Minderhedenbeleid. The Hague: SDU; 1994.
- Tweede Kamer, 1993–1994, kamerstuknummer 23684, ondernummer 2. Contourennota Integratiebeleid Etnische Minderheden. The Hague: SDU; 1994.
- Tweede Kamer, 1994–1995, kamerstuknummer 23409, ondernummer 16. Volksgezondheidsbeleid in een multiculturele samenleving. The Hague: SDU; 1995.
- Keij I. Standaarddefinitie allochtonen. Central Bureau of Statistics Magazine Index. 2000; 10: 24–25. Available online: http://www.cbs.nl/nr/rdonlyres/26785779-aafe-4b39-ad07-59f34dcd44c8/0/index1119.pdf.
- Van Mechelen P, Nieuwenhuizen P. De Allochtone zorgconsulent handboek. Utrecht: Forum; 2002. Available in print: Koninklijke Bibliotheek The national library of the Netherlands. https://www.kb.nl.
- Kamervragen met antwoorden TK 2010–2011 [Questions from parliament member Gerbrand and answers from Health Minister Schipper]. Tweede Kamer, 2010–2011, 2913, vraagnummer 2011Z10372. The Hague: SDU 2011.
- Klinische Genetica Nederland. Brief genetica en etniciteit (2008). https://zoek.officielebekendmakingen.nl/kst-27428-114-b1.pdf. Accessed 9 May 2014.
- Centrum voor Ethiek en Gezondheid. Briefadvies genetische aanleg en registratie van etniciteit (2011). http://www.ceg.nl/publicaties/bekijk/briefadvies-genetische-aanleg-en-registratie-van-etniciteit. Accessed 9 May 2014.
- Tweede Kamer, 2007–2008, kamerstuknummer 27428, ondernummer 114. Beleidsnota Biotechnologie. The Hague: SDU; 2008.
- Tweede Kamer, vergaderjaar 2011–2012, kamerstuknummer 27428, ondernummer 207. Beleidsnota Biotechnologie. The Hague: SDU; 2011.

## Appendix 2

### Original texts in Dutch

A. [Ten aanzien van de buitenlandse werknemers, die doorgaans slechts korte tijd in ons land zullen verblijven, zal het accent zelfs voornamelijk op het behoud van eigen identiteit worden gelegd. Heraanpassings moeilijkheden bij terugkeer naar eigen land zullen dan zo gering mogelijk zijn] (Kamerstuk Tweede Kamer 1969–1970 kamerstuknummer 10504 ondernummer 2).
B. [Ook voor de gezondheidszorg geldt als uitgangspunt dat leden van minderheidsgroepen op voet van gelijkheid toegang dienen te hebben tot de voorzieningen. […]. Dat betekent dat het voor de toegankelijkheid van de algemene voorzieningen wezenlijk is de deskundigheid van de hulpverleners ten aanzien van deze patiëntengroepen te vergroten. Hiervoor heeft het Ministerie van Welzijn, Volksgezondheid en Cultuur een programma ontwikkeld dat thans de volgende elementen omvat. In de eerste plaats is in 1982 het bulletin «Gezondheidszorg voor de buitenlandse patiënt» van de Geneeskundige hoofdinspectie in een oplage van 35000 stuks onder alle artsen, verloskundigen, wijkverpleegkundigen en instellingen voor gezondheidszorg verspreid] (Tweede Kamer, zitting 1982–1983, kamerstuknummer 16102, ondernummer 20–21).
C. [Als leidend beginsel voor een nieuwe visie op de aanwezigheid van personen uit diverse culturen in Nederland–of zij nu nieuwkomer zijn of al langer in Nederland verblijven–hanteert het kabinet het begrip burgerschap. […]. Burgerschap impliceert voor alle bij het integratieproces betrokkenen een keuze voor een blijvende deelname aan de Nederlandse samenleving met alle daaraan verbonden rechten en plichten] (Tweede Kamer, 1993–1994, kamerstuknummer 23 684, ondernummer 2).
D. [Deelt u tevens de mening dat het aanstellen van een allochtone zorgconsulent een verkeerd signaal afgeeft, namelijk dat de medische wereld zich aan de allochtoon aanpast in plaats van andersom] (Kamervragen met antwoorden TK 2010–2011. Tweede Kamer, 2010–2011, 2913, vraagnummer 2011Z10372).
E. [Ik ben van mening dat premiegelden zoveel mogelijk moeten worden besteed aan het verlenen van zorg. Het jezelf verstaanbaar maken is een verantwoordelijkheid van mensen die zich hier vestigen. Door als overheid en instelling steeds opnieuw de oplossingen aan te dragen waardoor de noodzaak van het leren van de taal veel minder is, wordt op den duur vooral ook de positie van de allochtonen zelf geschaad] (Kamervragen met antwoorden TK 2010–2011. Tweede Kamer, 2010–2011, 2913, vraagnummer 2011Z10372).
F. [Ik heb mij inmiddels nader geïnformeerd, onder andere bij de Vereniging van Klinische Genetica Nederland (VKGN) (zie bijlage)1 inzake een mogelijke betekenis–zoals verondersteld in de Trendanalyse–voor de gezondheidszorg van een eventuele relatie tussen genetica en etniciteit. De VKGN komt tot de conclusie dat etniciteit een factor kan zijn bij wetenschappelijk onderzoek, genetische diagnostiek en genetisch bevolkingsonderzoek] (Beleidsnota Biotechnologie. Tweede Kamer, 2007–2008, kamerstuknummer 27 428, ondernummer 114).

## Abbreviations

BVGB: Bureau Voorlichting Gezondheidszorg Buitenlanders [Bureau Health Education Healthcare for Foreigners]
CBS: Central Bureau of Statistics
NIGZ: National Institute for Health Promotion and Disease Prevention
OMVBW: Overlegorgaan Medische Verzorging Buitenlandse Werknemers [Consultation Body Medical Care Foreign Employees]
STS: Science and Technology Studies
VETC: Voorlichting eigen taal en cultuur [health education in own language and culture]
VKGN: Organisation for Clinical Genetics Netherlands
VWS: Ministry of Health, Welfare, and Sport

## Footnotes

1 This refers to a joint report written by Commissie Biotechnologie bij Dieren, Commissie Genetische Modificatie, and the Gezondheidsraad which discusses trends in technology which the authors of the report anticipate that the Dutch government will be confronted with in the near future.

2 MIKADO landelijke kenniscentrum voor interculturele zorg closed its doors in 2007; Forum closed in 2015; NIGZ closed in 2012; Overlegorgaan Gezondheidszorg en Multiculturele Samenleving OMG closed in 2005; Projectorganisatie Interculturalisatie van de Gezondheidszorg was dismantled in 2003.
